# Kinetics of the Equid Herpesvirus 2 and 5 Infections among Mares and Foals from Three Polish National Studs

**DOI:** 10.3390/v14040713

**Published:** 2022-03-29

**Authors:** Karol Stasiak, Magdalena Dunowska, Jerzy Rola

**Affiliations:** 1 Department of Virology, National Veterinary Research Institute, 24-100 Pulawy, Poland; karol.stasiak@piwet.pulawy.pl; 2 School of Veterinary Science, Massey University, Palmerston North 4442, New Zealand; m.dunowska@massey.ac.nz

**Keywords:** equid herpesvirus, EHV-2, EHV-5, foals, mares, viral load

## Abstract

Equid herpesvirus 2 (EHV-2) and 5 (EHV-5) are two γ-herpesviruses that are commonly detected from horses worldwide, based on several cross-sectional molecular surveys. Comparatively few studies examined the dynamics of γ-herpesvirus infection over time in a group of horses. The aim of the current study was to investigate the dynamics of EHV-2/5 infections among mares and their foals at three Polish national studs with different breeds of horses: Arabians, Thoroughbreds and Polish Konik horses. Nasal swabs were collected from each of 38 mare-foal pairs monthly for a period of 6 to 8 months. Virus-specific quantitative PCR assays were used to determine the viral load of EHV-2 and EHV-5 in each sample. All 76 horses sampled were positive for EHV-2 or EHV-5 on at least one sampling occasion. The majority (73/76, 96%) were infected with both EHV-2 and EHV-5. In general, the mean load of viral DNA was higher in samples from foals than from mares, but similar for EHV-2 and EHV-5 at most sampling occasions. There was, however, a considerable variability in the viral DNA load between samples collected at different times from the same foal, as well as between samples from different foals. The latter was more apparent for EHV-2 than for EHV-5. All foals became infected with both viruses early in life, before weaning, and remained positive on all, or most, subsequent samplings. The virus shedding by mares was more intermittent, indicating the existence of age-related differences. Overall, the data presented extend our knowledge of EHV-2/5 epidemiology among mares and foals.

## 1. Introduction

Horses are natural hosts to two γ-herpesviruses: equid herpesvirus type 2 (EHV-2) and equid herpesvirus type 5 (EHV-5). Equid γ-herpesviruses have been detected from horses worldwide with a higher frequency of detection in young horses compared to older ones [1,2,3]. However, the impact of these infections on the horse industry has been difficult to establish, as both viruses have been identified in samples from clinically normal horses, as well as from horses with respiratory disease [2,4,5,6,7]. Clinically affected horses typically show respiratory disease of various severity, with or without lymphadenopathy [8]. Some authors described a higher frequency of detection of EHV-2/5 from tracheal washes of horses affected by airway inflammation than from healthy horses, with or without overt disease, which led to the suggestion that EHV-2/5 infections may contribute to poor performance in horses [9,10,11]. However, results of other studies failed to demonstrate such a relationship [12,13]. The discrepancies between various investigations into the role of EHV-2/5 in equine respiratory disease are likely to reflect differences in variables such as the type of samples collected, timing of sampling, sensitivity and specificity of laboratory methods employed for processing of samples, definition of respiratory disease used, or the age-structure of the populations sampled. Herpesviruses are known for their ability to modulate the immune response of the host reviewed in [8,14]. Such modulation may predispose to secondary infections, and hence the clinical presentation may reflect the type of secondary pathogens that circulate in each population rather than clinical disease associated with primary EHV-2/5 infection [15]. It has also been suggested that EHV-2-induced disease is a consequence of an inappropriate immune response of the host to the viral infection, similarly to what has been described for infectious mononucleosis in humans [16]. Equid γ-herpesviruses display marked genomic heterogeneity [17,18,19], and hence it may be that EHV-2/5 variants with different pathogenic potential exist.

Equid γ-herpesviruses have also been detected from conjunctival swabs of horses with keratoconjunctivitis [20,21], as well as clinically normal horses [22,23], and EHV-5 has been implicated as an etiological agent in rare fibrotic lung disease of older horses with a fatal outcome [24].

Although EHV-2/5 infections are common among horses, the relative frequency of detection varied between studies, with EHV-2 [25,26,27] or EHV-5 [2,28,29,30,31,32] being most common. Most epidemiological studies comprised cross-sectional surveys of selected equine populations, with considerably fewer investigations of the shedding of EHV-2/5 over time. The available data suggest that foals become infected with EHV-2 and EHV-5 early in life and shed the virus for weeks to months in their nasal secretions [19,33]. Following primary infection, equid γ-herpesviruses establish lifelong latency in the lymphoid and neural tissues [34,35,36], with subsequent periodic recrudescence. The latter is believed to be associated with immunosuppression due to a variety of reasons such as disease or stress, but the exact triggers and mechanisms of recrudescence have not been elucidated.

The purpose of the current study was to assess the dynamics of γ-herpesvirus infections among mare-foal pairs of three Polish national studs.

## 2. Materials and Methods

### 2.1. Source of Samples

A total of 76 healthy mares and their foals from three national horse studs were included in the study (Table 1). Breeds included Polish Konik horse (Stud I, *n* = 28), Arabian (Stud II, *n* = 18) and Thoroughbred (Stud III, *n* = 30). Each stud was visited monthly, with the exception of two visits to Stud III, which were carried out at two monthly intervals. Samples were collected from all mares and their foals available on the visit day. The Arabian and Thoroughbred foals were kept with their dams in individual stalls, with some turnout time together in a shared paddock during the day. The Arabian foals (Stud II) were weaned either in September or in October 2015, when they were between six and eight months old. The Thoroughbred foals (Stud III) were weaned in July, August, or October 2016 when they were between five and seven months old. The weanlings at both studs were kept in individual stalls with some turned-out time as a group during the day. Most (*n* = 10) of the Polish Konik mare-foal pairs were housed together as part of a large group that was either kept in an open-plan barn or turned out in a shared paddock. The remaining Polish Konik mare-foal pairs (*n* = 4) were bred and managed in the open reserve under conditions as close to the natural environment as feasible. Foals from the barn/paddock group were weaned in November 2016, when they were between seven and nine months of age, foals from the open reserve group were not weaned during the study. The weaned foals and the mares were kept as two separate groups in the open-plan barn with some turn-out time during the day in their respective weanlings or mare groups.

### 2.2. Processing of Samples

Nasal swabs (14 cm length) were collected into a commercial universal transport medium (Copan Universal Transport Medium–UTM™, Brescia, Italy) and transported to the Department of Virology of the National Veterinary Research Institute in Pulawy (Poland) on ice packs within 6 h from collection. Upon arrival, each sample was vortexed in situ to release viruses into the transport medium and the swab was then discarded. An aliquot of each nasal swab sample was then transferred into an Eppendorf tube and stored at −80 °C for further processing. Total DNA was extracted from 200 µL of each sample, using the QIAamp DNA Mini Kit (Qiagen, Hilden, Germany) following the manufacturer’s protocol, and eluted with 50 µL of the supplied elution buffer. All extraction runs included positive (EHV-2 VR-701, ATCC) and negative (EHV-2/5 negative transport medium) extraction controls.

### 2.3. Real-Time PCR Assays

Primers targeting a conserved region of the *glycoprotein B* (*gB*) gene were used for the quantitative PCR (qPCR) assays for EHV-2 and EHV-5, as described elsewhere [3]. Each virus-specific qPCR reaction (25 µL) consisted of forward and reverse primers (Table 2) at a final concentration of 400 nM each, 200 nM of the probe and 2 µL of template DNA in 1× TaqMan^®^ Universal PCR Master Mix (Thermo Fisher Scientific, Warrington, UK). The samples were subjected to uracyl-DNA glucosylase treatment at 50 °C for 2 min, initial denaturation at 95 °C for 10 min, followed by 45 cycles of 94 °C for 10 s and 52 °C for 30 s. Negative (water) and positive (either EHV-2 VR-701 DNA or EHV-5 DNA extracted from a field virus, confirmed by sequencing) controls were included in each run. All reactions were performed using a LightCycler^®^ 96 System (Roche, Mannheim, Germany).

### 2.4. Quantification

In order to construct a standard curve for quantification of viral load, serial dilutions of a stock solution containing either a plasmid (EHV-2, 10^6^ to 10^0^ copies/µL) or gel-purified (QIAquick Gel Extraction Kit, Qiagen, Hilden, Germany) 518 bp PCR product that had been amplified using primers spanning the sequence targeted by EHV-5 qPCR and confirmed by sequencing (9.0 × 10^7^ to 9.0 × 10^0^ copies/µL) were used. To transform Cq values obtained in each qPCR assay to number of viral copies, we used the formula: Amount = 10^((Cq-b)/m)^, where m is the slope and b is the intercept from the regression equation. Samples with the Cq values higher than 40.3 and 36.5 were considered positive for EHV-2 and EHV-5 assays, respectively.

### 2.5. Data Analysis

Statistical analysis and graphs were performed using GraphPad Prism version 9.3.1 (GraphPad Software, San Diego, CA, USA, www.graphpad.com (accessed on 16 December 2021)). Descriptive statistics were used to summarize the data. Wilcoxon matched-pairs signed-rank test was used to compare viral load in nasal swabs between mares and foals, median EHV-2 with median EHV-5 loads at each stud on each sampling time, and median EHV-2/5 loads over time. The median viral loads from each foal obtained over multiple samplings during the period of study were compared to each other using Kruskal-Wallis test. For statistical analysis, the significance level was set at *p* < 0.05.

## 3. Results

### 3.1. EHV-2/5 Infections among Mares and Foals

**Figure 1:**
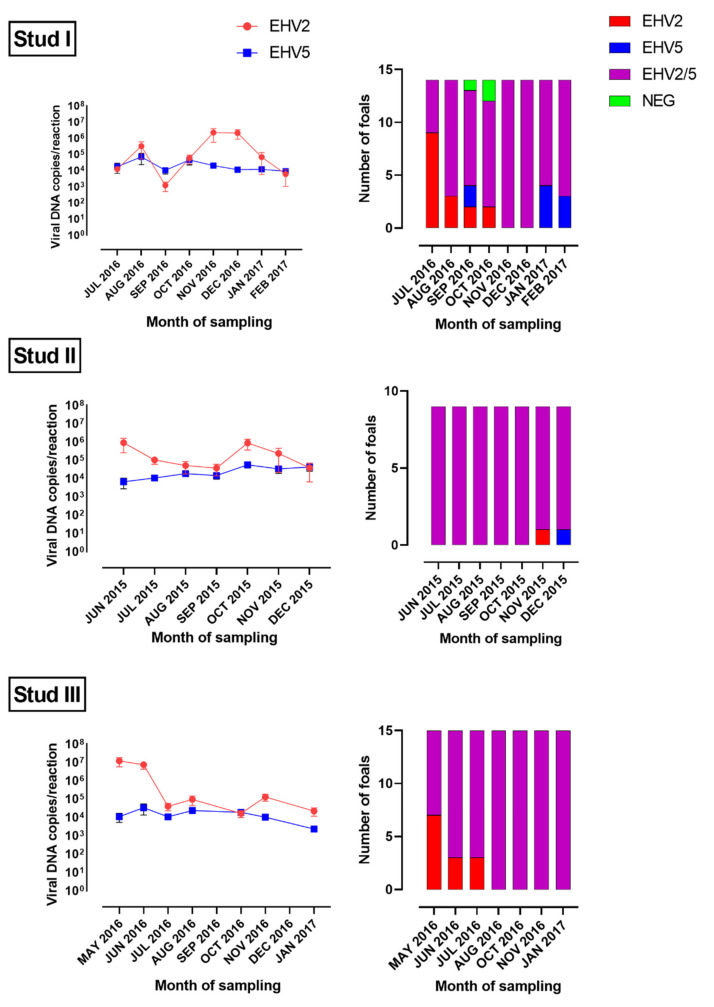
Number of foals positive for equid herpesvirus type 2 (EHV-2), equid herpesvirus type 5 (EHV-5) or both viruses at each sampling time (right). The corresponding mean load of EHV-2 and EHV-5 in nasal swabs from virus-positive foals is shown on the left. Error bars indicate standard deviation.

**Figure 2:**
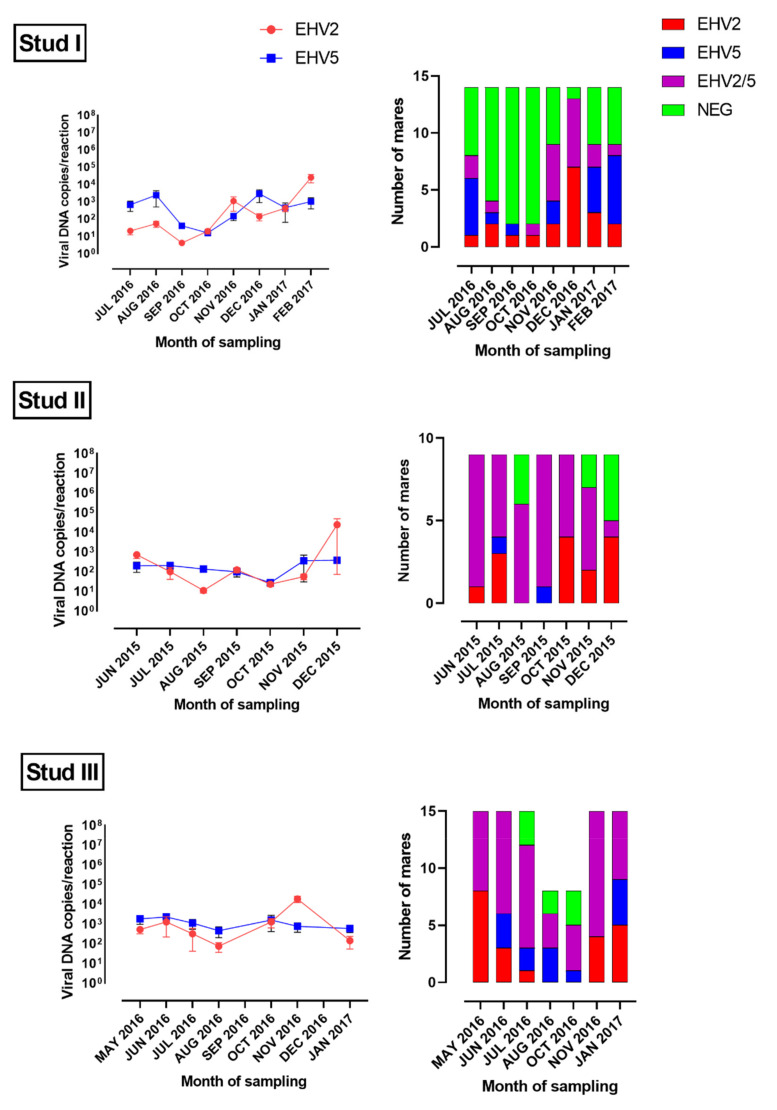
Number of mares positive for equid herpesvirus type 2 (EHV-2), equid herpesvirus type 5 (EHV-5) or both viruses at each sampling time (right). The corresponding mean load of EHV-2 and EHV-5 in nasal swabs from virus-positive mares is shown on the left. Error bars indicate standard deviation.

**Figure 3:**
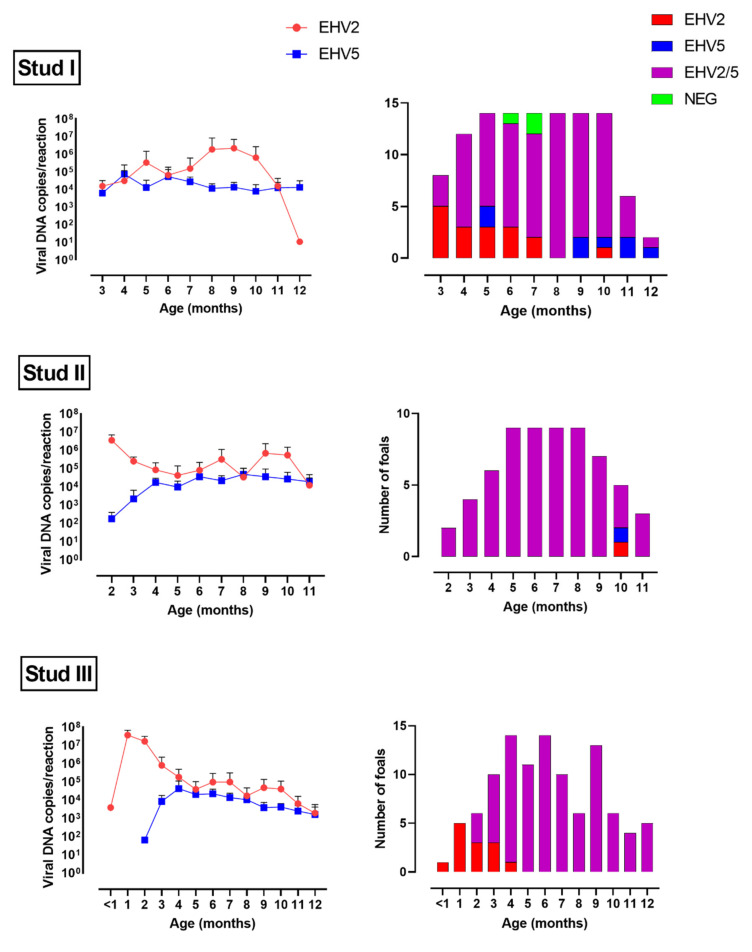
Number of foals positive for equid herpesvirus type 2 (EHV-2), equid herpesvirus type 5 (EHV-5) or both viruses stratified by age (right). The corresponding mean load of EHV-2 and EHV-5 in nasal swabs from virus-positive foals is shown on the left. Error bars indicate standard deviation.

Altogether, EHV-2 was detected in 426 swabs from 75 horses, and EHV-5 from 386 swabs from 74 horses (Figure 1 and Figure 2, Supplementary Table S1). All 76 horses (38 mares and 38 foals) sampled were positive for EHV-2 or EHV-5 on at least one sampling occasion. The majority (73/76, 96%) were infected with both EHV-2 and EHV-5. Two horses (M2 and M13 from Stud I) were positive for EHV-2 only and one horse (M6 from Stud I) for EHV-5 only. Foals became infected with both viruses early in life, before weaning. All foals from Stud II were positive for both EHV-2 and EHV-5 at the first sampling occasion (June 2015) when they were between 2- and 5-month-old (Figure 3). Some foals at Studs I and III became infected with EHV-2 earlier than with EHV-5. However, by the age of five months (Stud III) or eight months (Stud I) all sampled foals were positive for both viruses (Figure 3).

### 3.2. Load of EHV-2 and EHV-5 DNA

**Figure 4:**
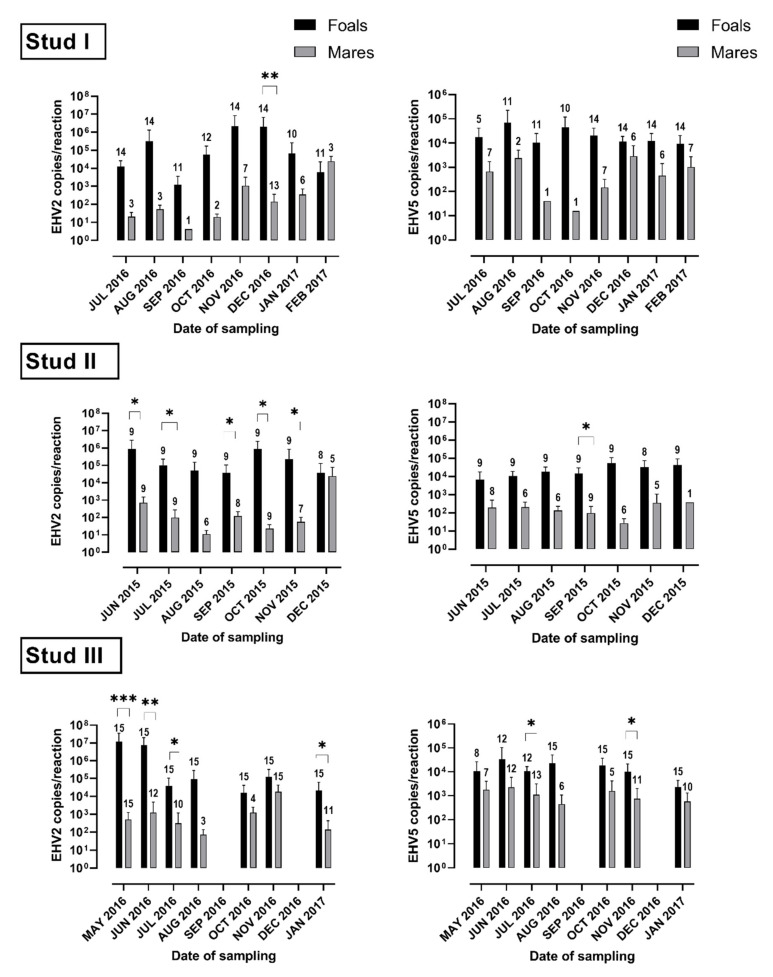
Comparison of the viral load of equid herpesvirus type 2 (EHV-2) and equid herpesvirus type 5 (EHV-5) DNA in nasal swabs from mares and from foals at each of the participating studs. The bars show mean copy numbers of viral DNA per reaction with standard deviation. The number of virus-positive horses at each sampling time is shown above the bars. Statistically significant differences in the mean viral load between mares and foals at the same sampling time (Wilcoxon matched-pair signed rank test) are indicated by * (<0.05), ** (<0.01) and *** (<0.001).

**Figure 5:**
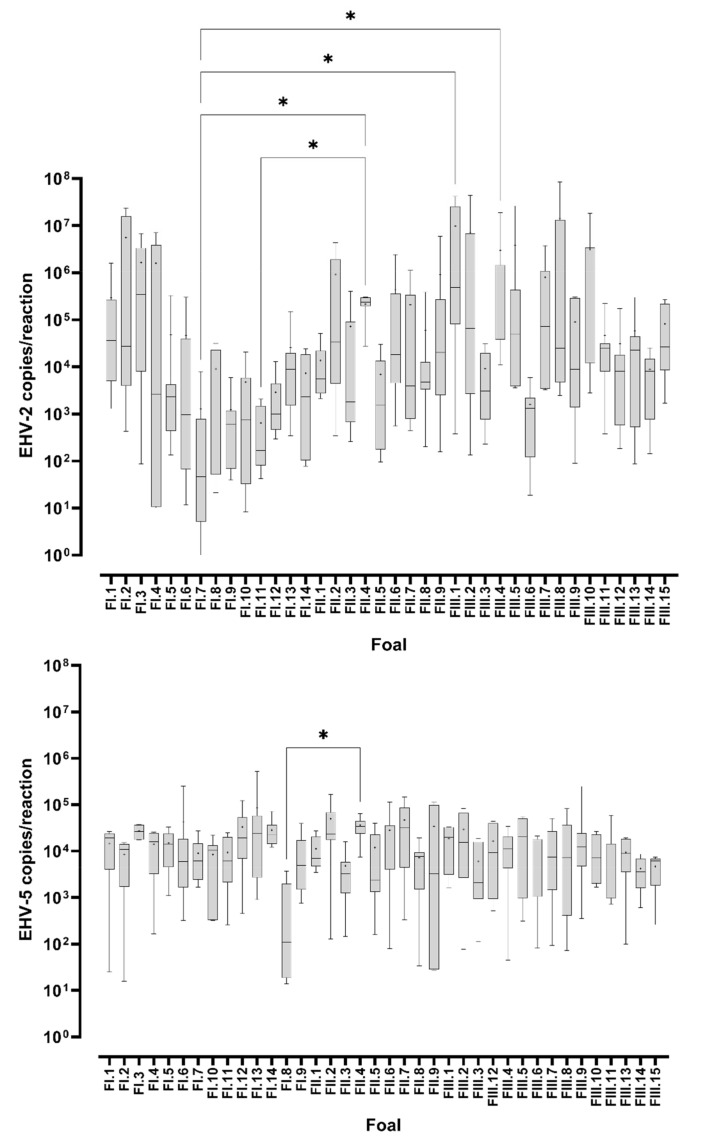
Variability of the viral load of equid herpesvirus type 2 (EHV-2) and equid herpesvirus type 5 (EHV-5) DNA in virus-positive nasal swabs from individual foals. The box-plots show median copy numbers of EHV-2 (top graph) and EHV-5 (bottom graph) DNA from multiple samplings over a period of eight months (Stud I), seven months (Stud II), or nine months (Stud III). * indicates significant difference between the medians (*p* < 0.05).

The mean load of viral DNA (copies/reaction) in virus-positive swabs was similar for EHV-2 and EHV-5 at most sampling occasions (Figure 1 and Figure 2). At some samplings (e.g., the first sampling at Studs II and III or Nov/Dec samplings at Stud I) the mean load of EHV-2 appeared to be higher than the mean load of EHV-5 for foals (Figure 1), but that difference was not statistically significant (*p* > 0.05). Similar results were obtained when the data was analyzed by the foals’ age (Figure 3). In general, the median load of viral DNA was higher in samples from foals than from mares for both EHV-2 and EHV-5 (Figure 4). There was, however, a considerable variability in the viral DNA load between samples collected at different times from the same foal, as well as between samples from different foals. The latter was more apparent for EHV-2 than for EHV-5 (Figure 5).

### 3.3. Shedding Pattern of EHV-2/5

The shedding pattern of EHV-2/5 differed between horses. The majority (7/9, 78%) of foals from Stud II tested positive for both EHV-2 and EHV-5 on all sampling occasions. The two remaining foals tested positive for both viruses on 6/7 sampling occasions, and positive for only one virus (either EHV-2 or EHV-5) on one sampling occasion each. Approximately half (7/15, 47%) of foals from Stud III tested positive for only EHV-2 during the first one to three samplings, but subsequently they became infected with EHV-5 and tested positive for both viruses until the end of the study. The remaining eight foals from Stud III tested positive for both EHV-2 and EHV-5 on all seven testing occasions. In contrast, only one foal (F13) from Stud I tested positive for both EHV-2 and EHV-5 on all eight sampling occasions. An additional three foals (F3, F8, F14) tested positive for EHV-2 on all sampling occasions but were positive for EHV-5 on only some of the samplings, and four foals (F5, F9, F11, F12) tested positive for EHV-5 on all sampling occasions, but were positive for EHV-2 on only some of the samplings. The remaining six foals from Stud I (F1, F2, F4, F6, F7, F10) were positive for EHV-2 or EHV-5 on some, but not all, sampling occasions. Similar to what was observed at Stud III, 9/14 (64%) foals from Stud I tested positive for EHV-2 only at the first sampling in July 2016 and became infected with EHV-5 between 1 and 4 months later. The shedding of both EHV-2 and EHV-5 by the mares was intermittent at all three studs. This was most apparent for Stud I, were percent of virus-positive mares varied from 14% (September and October 2016) to 93% (December 2016) (Figure 2).

## 4. Discussion

The aim of the study was to determine the kinetics of EHV-2 and EHV-5 infections at three horse studs in Poland. We have followed 38 mare-foal pairs over 6 to 9 months. To our knowledge, this is the largest prospective study of equid γ-herpesvirus epidemiology at breeding stud farms thus far.

All mares and their foals were positive for EHV-2 or EHV-5 on at least one sampling occasion, indicating that equid γ-herpesvirus infections were common among horses from all three groups sampled. This is consistent with the results of a previous Polish study where 77.2% and 47% of 540 foals and horses of various ages sampled on a single occasion were positive for EHV-2 and EHV-5, respectively [37], as well as with data from other countries [6,25,38,39].

While all mares were positive for EHV-2 or EHV-5 at least once, the proportion of EHV-2/5 positive mares on most sampling occasions was lower at Stud I than at Studs II and III (Figure 2). This seems to support results of a previous study, where Polish Konik horses were less likely to be shedding EHV-2 or EHV-5 than Thoroughbred or Arabian horses [37]. Polish Konik horse is a native primitive breed that exhibits some physical characteristics of now extinct equid subspecies *Tarpan* [40]. It could be hypothesized that Polish Konik horses can better control EHV-2/5 infection than horses from other breeds, possibly due to differences in their genetic background, but such a hypothesis would need to be tested in further experiments. It is equally possible that other variables such as management factors or environmental conditions influenced the data. In addition, a comparatively small number of mare-foal pairs sampled at each stud precludes extrapolation of these findings beyond the sampled populations.

Consistent with previous reports [6,38,41], foals in the current study remained positive for EHV-2 and EHV-5 on all, or most, subsequent samplings after the initial infection. The virus shedding by mares, particularly those from Studs I and III, was more intermittent, indicating the existence of age-related differences. Based on these findings, the assessment of the EHV-2/5 infection status of mares (and possibly other adult horses) should not rely on a single sample.

It remains to be established whether repeated detection of EHV-2/5 represented reactivation of a latent virus, persistent infection, or re-infection with different variants of EHV-2/5 that circulated within each population of horses. The latter may be supported by the existence of a considerable heterogeneity among both EHV-2 and EHV-5 [6,17,19,33]. Molecular analysis of genomic variants present in each sample would help to differentiate between these possibilities. Such work is currently underway and will be reported separately.

Foals at all three studs became infected with both EHV-2 and EHV-5, early in life, by the time they reached five months of age. The exact timing of infection with either virus could not be established, as all foals were positive for EHV-2, and most were also positive for EHV-5, at the first sampling. In addition, only a small number of samples from foals younger than three months were available. However, detection of the viruses in nasal swabs of foals as young as one to two months is consistent with data reported by others, with some reports of EHV-2 infection occurring within the first two weeks of life [6,17,39,41,42,43]. It has been suggested that the source of EHV-2 infection in such young foals is most likely their dams [6,41]. However, in the current study, both the level and the frequency of shedding of γ-herpesviruses were comparatively lower among mares than among foals (Figure 4). Combined with the fact that foals at each stud had an opportunity to interact with each other, it is likely that transmission between foals contributed to the high rates of infection observed. This is supported by results of other studies where virus genotypes from some foals were more similar to genotypes from other foals than to those from their dams for both EHV-2 [42] and EHV-5 [44].

Several foals from Studs I and III were infected with EHV-5 later than with EHV-2. A similar trend was not observed at Stud II, possibly due to a low number of horses younger than four months sampled at that stud. The reasons for the later timing of EHV-5 infection compared with EHV-2 infection among some foals are unclear, but similar findings have been reported previously [6,25,38,43]. It is possible that earlier infection with EHV-2 reflects higher levels of EHV-2 shedding, as compared with EHV-5, early in life. Although not statistically significant, this seemed to be a trend observed in the current study for one- to three-month-old foals from Studs II and III, but not for foals from Stud I, possibly since the latter were first sampled at the age of three months. We are aware of only one other longitudinal study where both EHV-2 and EHV-5 viral loads were quantified in nasal swabs from 30 Icelandic mare-foal pairs followed monthly [43]. In that study, the mean load of EHV-2 in nasal swabs was highest when the foals were two to four months of age, while the mean load of EHV-5 increased slowly for the first five months of age without reaching the levels of EHV-2 loads during the foals’ first six months of life [43]. In contrast, the load of EHV-5 remained relatively constant over time for foals from all three studs in the current study, with similar levels of EHV-2 and EHV-5 shed by Stud II and III foals from four months of age until the end of the study. Others also reported similar mean loads of EHV-2 and EHV-5 in nasal secretions from foals [3], but that was based on data from a cross-sectional study where all foals were considered together as one age-group.

The levels of EHV-2 shed by foals from Stud I were similar to the levels of EHV-5 when the foals were three and 4 months old, but about 2 logs higher when they were eight- to ten months old (Figure 3). Results from two other studies that focused on EHV-2 only seem similar to those reported here for studs II and III. The mean load of EHV-2 was highest among one- to three-month-old foals followed on a monthly basis in one study [42] and the highest levels of EHV-2 were shed in nasal secretions of three-month-old foals compared with the same foals at the age of one and five months in another study [17]. Overall, foals at all three studs shed large quantities of EHV-2 and EHV-5 in their nasal secretions within the first year of life. The relative load of the two viruses differed across ages and between populations sampled, but EHV-2 infection tended to be more frequent, often with higher levels of shedding, within the first three months of life.

The load of both EHV-2 and EHV-5 in nasal swabs varied not only between samples collected from the same horse over time, but also between samples collected from different horses throughout the study period, with some low and high-shedders. This was more apparent for EHV-2 than for EHV-5 (Figure 5). It seems logical to assume that the load of the virus shed was controlled, at least in part, by the immune response to infection. Detection of EHV-2/5 from foals as young as one to two months of age suggests that equine γ-herpesvirus infection occurred in the presence of maternal antibody. Although the correlates between EHV-2/5 infection and protection are currently poorly understood, it may be that antigen-specific mucosal antibodies are more important in protection than systemic antibodies acquired through the intake of colostrum. However, Thorsteinsdottir et al. [43] reported that the peak EHV-2 shedding coincided with a decline of the EHV-2/5 maternal antibody, and that the load of EHV-2 in nasal secretions decreased as the levels of EHV-2/5 IgG increased. By extrapolation, it is possible that the differences in the levels and pattern of EHV-2 shedding by foals in the current study reflected the differences in the levels of EHV-2/5 maternal antibody early in life, but we cannot confirm this as the latter was not measured. In contrast to EHV-2, the mean load of EHV-5 rose in parallel to the rise in the EHV-2/5 specific IgG in the Iceland-based study [43], while it remained relatively constant in the current study. Altogether, there appear to be differences between the epidemiology of EHV-2 and EHV-5 among foals from all three studs. These are likely to be related to the interactions between these viruses and the immune system, which should be addressed in future studies.

The effect of EHV-2/5 infection on health status of the horses was not the focus on the current study. However, none of mares and foals sampled showed overt respiratory disease at the time of sampling. This suggests the EHV-2/5 infection was not associated with clinical disease among the sampled population. Alternatively, the presence of disease may have been missed if it occurred in-between the sampling visits or if it was mild or subclinical. Detection of the latter would have required additional diagnostic modalities such as endoscopy.

Herpesvirus reactivation has been anecdotally linked to stress, although experimental data to support such a link for equine herpesviruses are somewhat conflicting [45,46]. Weaning is considered to be a stressful event for both foals and their dams [47,48]. As such, it would be reasonable to expect an increase in the rate of EHV-2/5 infections or reactivations during a post-weaning period. This may have been one explanation for the fact that mares from Stud I showed the highest rate of EHV-2/5 shedding during the first post-weaning visit (December 2016). One limitation of the study was the fact that samples were not collected within the first few days post-weaning when the foals and mares would be expected to be the most stressed, which may explain the lack of any clear impact of weaning on the frequency or levels of EHV-2/5 shedding at other studs. In addition, foals from all three studs as well as most mares from Studs II and III had been already actively infected with EHV-2/5 by the time the foals were weaned.

In summary, some common features of equid γ-herpesvirus epidemiology were evident at all three breeding farms included in the study. Infections with both EHV-2 and EHV-5 were common among mares and foals sampled. Foals became infected with both viruses early in life and shed higher amounts of EHV-2 and EHV-5 than mares. The shedding by the mares was more intermittent than that by the foals. There was a considerable variability in the viral DNA load between individual foals and between the same foals sampled at different times. The data presented adds to our knowledge of the epidemiology of EHV-2/5 at the horse breeding farms.

## Figures and Tables

**Table 1:** Source and number of nasal swabs collected from mares and their foals at three different Polish national studs.

Stud	Breed	No of Mare-Foal Pairs	No of Samples	No of Visits	Dates
Stud I	Polish Konik	14	224	8	July 2016–February 2017
Stud II	Arabian	9	126	7	June 2015–December 2015
Stud III	Thoroughbred	15 *	194	7	May 2016–January 2017
	Total	38	544	22	

* In August and October, seven and eight mares, respectively, were away from the stud and hence not available for testing.

**Table 2:** Primers and probes used for the detection and quantification of EHV-2 and EHV-5.

Virus	Primers/Probes (5′ to 3′)	Size (bp)
EHV-2	Forward: GTGGCCAGCGGGGTGTTC	78
	Reverse: CCCCCAAAGGGATTYTTGAA	
	Probe: FAM-CCCTCTTTGGGAGCATAGTCTCGGGG-TAMRA	
EHV-5	Forward: AACCCGCCGTGCATCA	66
	Reverse: AGGCGCCACACACCCTAA	
	Probe: FAM-ACAACACCACCAACCCCTTTCTGCTG-TAMRA	

## Data Availability

Detailed data for all horses sampled are provided in the supplementary file.

## Supplementary Materials

The following supporting information can be downloaded at: https://www.mdpi.com/article/10.3390/v14040713/s1, Table S1. Detailed data for all horses sampled in the study.
